# Characterisation of a tripartite α-pore forming toxin from *Serratia marcescens*

**DOI:** 10.1038/s41598-021-85726-0

**Published:** 2021-03-19

**Authors:** Alicia M. Churchill-Angus, Thomas H. B. Schofield, Thomas R. Marlow, Svetlana E. Sedelnikova, Jason S. Wilson, John B. Rafferty, Patrick J. Baker

**Affiliations:** 1 Department of Molecular Biology and Biotechnology, University of Sheffield, Firth Court, Western Bank, Sheffield, S10 2TN South Yorkshire UK; 2 Current address: Astbury Centre for Structural and Molecular Biology, University of Leeds, Leeds, LS2 9JT UK

**Keywords:** Structural biology, Structural biology, Biochemistry, Proteins, Membrane proteins, Microbiology, Bacteria, Bacterial toxins

## Abstract

Tripartite members of the ClyA family of α-PFTs have recently been identified in a number of pathogenic Gram-negative bacteria, including the human pathogen *Serratia marcescens*. Structures of a Gram-negative A component and a tripartite α-PFT complete pore are unknown and a mechanism for pore formation is still uncertain. Here we characterise the tripartite SmhABC toxin from *S. marcescens* and propose a mechanism of pore assembly. We present the structure of soluble SmhA, as well as the soluble and pore forms of SmhB. We show that the β-tongue soluble structure is well conserved in the family and propose two conserved latches between the head and tail domains that are broken on the soluble to pore conformational change. Using the structures of individual components, sequence analysis and docking predictions we illustrate how the A, B and C protomers would assemble on the membrane to produce a complete tripartite α-PFT pore.

## Introduction

The Gram negative bacteria *Serratia marcescens* is widespread throughout the environment, with strains involved in marine, plant and animal infections^1–3^. Multiple strains have been identified as important antibiotic resistant nosocomial human pathogens^2^, and as the causal agent in a number of infections including respiratory, urinary tract, septicaemia and meningitis^1,4^. Conversely, other plant-associated strains have been reported to promote plant growth and protect plants from infection by the production of antifungal and antibacterial compounds^5^.

Recently a number of strains of *S. marcescens,* including plant-associated strain MSU97^5^ and human pathogenic strain UMH7^6^, have been identified as containing homologues of the tripartite pore-forming toxin (PFT) AhlABC from *Aeromonas hydrophila*^7^. Tripartite α-PFTs are members of the ClyA α-PFT family^8^. The ClyA α-PFT family contains pores composed of one (*E.coli*, ClyA)^9–11^, two (*Yersinia enterocolitica*, YaxAB and *Xenorhabdus nematophila*, XaxAB)^12,13^ or three (*A. hydrophila*, AhlABC and *Bacillus cereus* NheABC and HblL_1_L_2_B)^7,8,14,15^ protein components. Proteins in this family undergo a large scale conformational change from a compact soluble structure, where the hydrophobic residues are hidden often within a β-tongue motif, to an extended pore structure with the hydrophobic residues exposed in two extended α-helices which insert into the cell membrane^7,9,12–14^. To form a complete oligomeric pore all ClyA α-PFT family members share three common features, which are carried on different components in the bipartite and tripartite pores. These are: a short single leaflet spanning hydrophobic helix-turn-helix motif that provides the initial membrane binding event, carried on the ClyA β-tongue motif; or by YaxA and XaxA, or AhlC, NheC and Hbl-B, in the single, bipartite and tripartite toxins, respectively; a longer membrane-spanning hydrophobic or amphipathic helical region (ClyA N-terminal helix; YaxB, XaxB; AhlB, NheB, HblL_2_); and finally a hydrophilic lining to the interior of the pore (ClyA N-terminal helix; YaxB, XaxB; AhlA, NheA, HblL_1_)^7^. In ClyA, and the bipartite pores YaxAB and XaxAB, this results in an oligomeric hydrophilic pore assembled from amphipathic helices, surrounded by the hydrophobic single leaflet anchoring components^10,12,13^. In addition to their haemolytic activity, recent studies of the Nhe and Hbl tripartite PFTs found in food poisoning strains of *B. cereus* show that both are able to activate the NLRP3 inflammasome leading to septic shock in mice, and inhibition of this response prevents lethality, demonstrating the importance of this toxin family in bacterial pathogenicity^16,17^. Understanding how these tripartite α-PFTs function is thus an important step in combatting infection and designing virulence targeted therapies.

Although significant progress has been made on structural studies of tripartite α-PFTs driven by the discovery of tripartite α-PFTs in Gram negative bacteria^7^, structures of the A component from a Gram negative species or the complete tripartite pore remain elusive. Structures of either of these would allow for a better understanding of how the A, B and C components fulfil the three features observed in other ClyA α-PFT family members. The high sequence similarity between the proteins in Gram negative tripartite PFTs means that the structure and function of the component proteins must be closely related across the different species^7^. As *S. marcescens* has implications in human infection and antibiotic resistance, together with its antimicrobial properties and prevalence in the environment, it is an ideal choice for further studies of tripartite PFTs.

Here we show that *S. marcescens* has a tripartite haemolytic α-PFT (SmhABC), and propose a mechanism of pore assembly. We present the structures of the soluble A component (SmhA), as well as the soluble and pore conformations of the B component (SmhB). We show how these structures share high structural similarity with other members of the ClyA α-PFT family, especially those of NheA and AhlB, and show that a chimeric Smh/Ahl pore retains full lytic activity. Using these structures along with those of the closely related *A. hydrophila* AhlABC toxin, we propose a mechanism of soluble to pore transformation, model membrane-associated complexes of the three proteins of the Gram negative tripartite α-PFTs and present a structure based model of the location of the A component in a tripartite PFT pore.

## Results

### SmhABC lyses erythrocytes and forms pores in erythrocytes and liposome membranes

**Figure 1:**
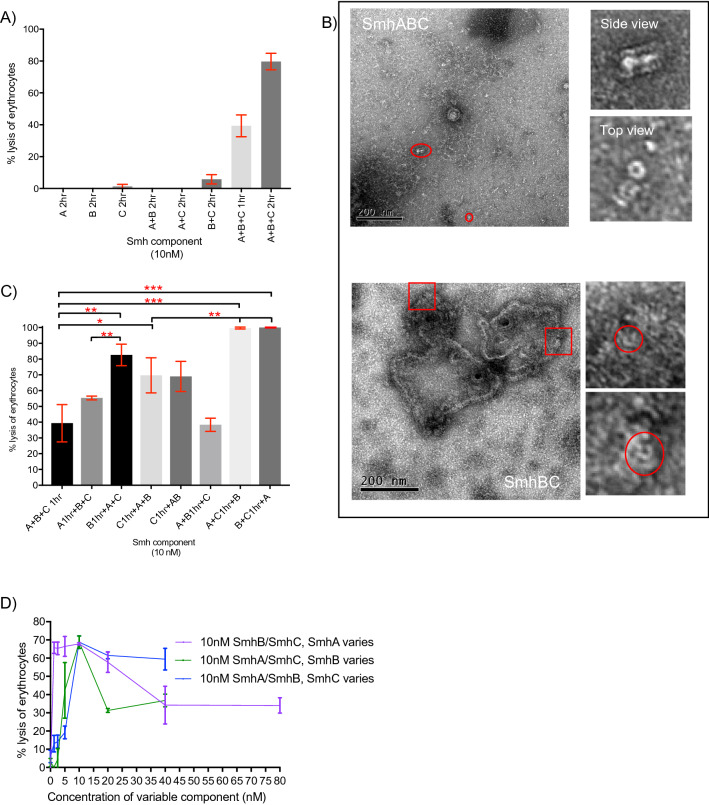
Lytic and pore forming activity of Smh toxin. (**A**) Percentage lysis of erythrocytes with each protein alone and in combination at a ratio of 1:1:1. (**B**) TEM negative stain images of erythrocytes incubated with SmhA + SmhB + SmhC (top), showing side and top views of pores in membrane fragments, and SmhB + SmhC (below) showing top views of pores which line the membranes of intact erythrocytes. (**C**) Percentage lysis after pre-incubation of erythrocytes for 1 h at 37 °C with either SmhA, SmhB or SmhC alone or together, before addition of the remaining components with a further 1 h incubation. Red stars above bars represent P value, * P ≤ 0.05; ** P ≤ 0.01; *** P ≤ 0.001. (**D**) Percentage lysis with varying concentrations of SmhA, SmhB or SmhC and fixed concentrations (10 nM) of the remaining 2 components. All assays were done in triplicate (n = 3) with error bars corresponding to the mean ± standard deviation.

Homology searches with *A. hydrophila* AhlABC have previously identified *S. marcescens* SmhABC as a possible tripartite α-PFT^7^. To confirm this assignment, SmhA, SmhB and SmhC were expressed, purified and used in haemolytic assays with erythrocytes. Individual components showed no lytic activity alone, neither did mixtures of SmhA + SmhB and SmhA + SmhC (Fig. 1A). However, incubating equimolar concentrations of SmhB and SmhC together with erythrocytes showed 5% ± 2% (S.D) lysis after 2 h, while SmhA, SmhB and SmhC together showed 40% ± 7% (S.D) lysis in 1 h and 80% in 2 h (Fig. 1A). Negative stain EM images of reaction mixtures for SmhA + SmhB + SmhC, and SmhB + SmhC showed that for SmhA + SmhB + SmhC, individual pores and pores bound to the membrane were present, while for SmhB + SmhC pores bound to membranes were visible, indicating that SmhABC is indeed an α-PFT (Fig. 1B). In the SmhABC images only small erythrocyte membrane fragments were visible, unlike the case with *A. hydrophila* AhlABC where large membrane fragments could be seen containing pore structures^7^. This is possibly due to the much greater erythrocyte lysis efficiency of SmhABC (40% lysis in 1 hr at 10 nM ) compared to AhlABC (95% lysis 1 hr, at 1 μM).

To determine an assembly order for the SmhABC pore, erythrocytes were pre-incubated with different components for 1 h before addition of the remaining components. Pre-incubation with either SmhB or SmhC resulted in 82 ± 6% (S.D) and 70% ± 10% (S.D) lysis after 1 h, respectively, double that of the mixture of SmhA + SmhB + SmhC without pre-incubation (p = 0.005, and p = 0.03) (Fig. 1C). When erythrocytes were pre-incubated with mixtures of SmhA + SmhC or SmhB + SmhC, 100% lysis was achieved in 1 h, significantly greater than with no pre-incubation (SmhA + SmhB + SmhC, p = 0.0009) and to the single components alone (p = 0.01 and p = 0.009) (Fig. 1C). Pre-incubation with SmhA or SmhA + SmhB followed by addition of the other components resulted in 55% ± 1% (S.D) and 40% ± 3% (S.D) lysis respectively, with no significant difference to the SmhA + SmhB + SmhC control with no pre-incubation (p = 0.08 and p = 0.90) (Fig. 1C). It thus appears that pre-binding of either SmhB or SmhC alone, or together, to the membrane increases pore efficiency, whereas SmhA only increases efficiency if SmhC is also present.

For many ClyA family α-PFTs, efficacy of the pore is controlled by regulating the formation of soluble off pathway complexes^14,18,19^. Lytic assays with varying concentrations of SmhA, SmhB or SmhC were used to study any effect of ratio on potency of the SmhABC system. When SmhA concentration was varied maximal lysis was achieved up to a 1:1:1 ratio (A:B:C), lysis then decreased steadily to a minimum of 34% ± 10% (S.D) at a ratio of 4:1:1 and remained at this value up to a ratio of 8:1:1 (Fig. 1D). When varying both SmhB and SmhC, lysis increased up to a maximum at a ratio of 1:1:1, however, while no reduction of activity was observed for SmhC at higher concentrations, lytic activity dropped to 30% ± 2% (S.D) at a ratio of 1:2:1 and remained at 30% lysis at higher concentrations of SmhB (Fig. 1D). This showed that both SmhA and SmhB have an inhibitory effect on lysis at high concentrations. Size exclusion analysis of soluble Smh proteins at 37 °C showed that mixtures of SmhB and SmhC form a large soluble complex (~ 890 kDa) while mixtures of SmhA and SmhC precipitate at 37 °C. In addition, SmhA, SmhB and SmhC together in solution formed a 1 MDa complex containing all three proteins (Supplementary Fig. 1). Incubation of this soluble SmhABC complex with erythrocytes showed no lysis after 1 h. Similar assays with the soluble SmhBC complex also showed no lysis, and addition of SmhA also resulted in no lysis after a further 1 h incubation. As both the soluble SmhABC and SmhBC complexes are both inhibitory to lysis, this suggests that the Smh pore efficacy is regulated by the formation of off pathway soluble complexes, as seen in other family systems from *Bacillus, Yersinia* and *Xenorhabdus*^7,13,18,20^. This control mechanism, however, does not involve any potential SmhA/SmhB complex, as none could be observed on gel filtration and there was no difference in lysis efficiency comparing pre-incubated erythrocytes with SmhC followed by addition of SmhA and SmhB, or premixing SmhA and SmhB for one hour before adding to cells pre-incubated with SmhC (p = 0.9411, Fig. 1C).

### Structure of SmhB

**Figure 2:**
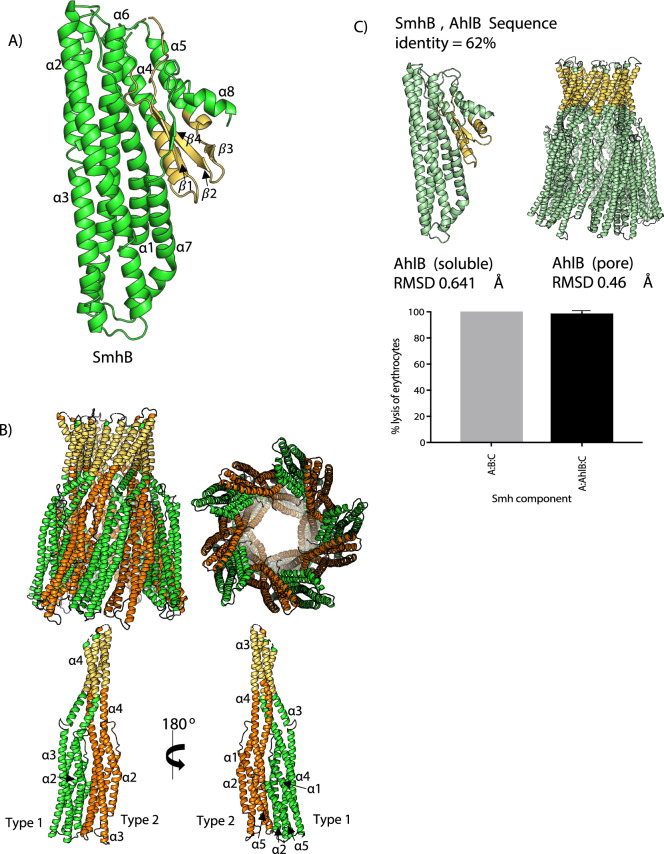
Structure of soluble and pore form SmhB. (**A**) Structure of soluble SmhB (PDB:6ZZH), the hydrophobic β-tongue (yellow) is buried from the solvent. (**B**) Pore Structure of SmhB (PDB:7AOG). 10 monomers of SmhB, in two conformations (type 1 – green, and type 2 – orange), assemble to form a helical pore with a hydrophobic head (yellow) made up of extended hydrophobic helices (top), with the individual type1-type2 dimers shown (bottom). (**C**) The structures of both soluble and pore SmhB are almost identical to those of AhlB (top), haemolytic assay results (below) for SmhABC and chimeric toxin, SmhA + AhlB + SmhC, showing both toxin combinations reach 100% lysis after 1 h incubation with erythrocytes.

The proteins of the Smh PFT system of *S. marcescens* share high levels of sequence identity with those of the Ahl system from *A. hydrophilia* (46, 62, 43% identity, respectively), however, activity of the Ahl PFT is not regulated by off pathway soluble complexes^7^. To further characterise the Smh PFT we determined the structure of SmhB to compare to AhlB. The soluble form of SmhB was solved at a resolution of 1.84 Å in space group P2_1_2_1_2_1_ by molecular replacement using the soluble form of AhlB^7^ (PDB code 6GRK) as a search model (Supplementary Fig. 2A) (Table 1). As expected, soluble SmhB folds into the family observed β-tongue structure, with a five-helix bundle tail (α1, α2, α3, α6, α7), and a head domain containing three α-helices (α4, α5, α8) alongside a four-strand β-sheet (β1, β2, β3, β4), which includes the β-tongue motif (β1 and β2). The head domain of SmhB is largely hydrophobic. This is particularly evident in the β-tongue and helices α4 and α5, which together constitute the predicted hydrophobic transmembrane region (G175—L233), (Fig. 2A). The crystal asymmetric unit contained two closely related monomeric AhlB molecules (RMSD Cα 0.56 Å) with the only significant differences in the tail domain, where α7 from chain A folds as a single helix with a kink at S320, while in chain B α7 is divided into α7a and α7b by a short loop region (T319-V321). Chain A will be described in this section unless otherwise stated.

**Table 1:** X-ray data collection and refinement statistics for Smh structures.

	SmhB soluble (PDB:6ZZ5)	SmhB soluble form2 (PDB:6ZZH)	SmhB pore (PDB:7AOG)	SmhA semet (PDB:7A26)	SmhA form2 (PDB:7A27)
**Data collection**					
Beamline	I04-1	I04-1	I24	I03	I04-1
Wavelength (Å)	0.9159	0.9159	0.9795	0.9792	0.9159
Space group	P2_1_2_1_2_1_	P2_1_	C2	P4_2_	P2_1_2_1_2_1_
**Cell parameters**					
a (Å) =	50.94	95.93	224.48	151.36	81.24
b (Å) =	113.95	49.73	118.06	151.36	92.58
c (Å) =	130.37	99.49	209.53	133.46	221.1
α (°) =	90	90	90	90	90
β (°) =	90	118.43	109.44	90	90
γ (°) =	90	90	90	90	90
Molecules per asymmetric unit	2	2	10	8	4
Resolution (Å)	1.84–65.18 (1.84–1.87)	1.86 – 87.51 (1.86–1.88)	6.98 – 66.77 (6.98 – 7.10)	2.98 – 67.69 (2.98 – 3.03)	2.57–110.55 (2.57–2.61)
Total reflections^a^	445,968 (15,821)	286,930 (8241)	28,759 (1072)	1,727,990 (88,850)	703,016 (35,733)
Unique reflections^a^	66,582 (3207)	50,372 (2519)	8305 (350)	61,518 (3065)	53,914 (2644)
*R*_merge_^a,b^	0.139 (1.214)	0.1 (0.899)	2.295 (3.384)	0.406 (3.048)	0.190 (2.129)
*R*_pim_^a,c^	0.078 (0.842)	0.044 (0.525)	1.403 (2.356)	0.078 (0.575)	0.055 (0.595)
Mean I/σ(I)^a^	9.5 (1.0)	10.4 (1.4)	1.3 (0.5)	7.2 (1.3)	8.5 (1.1)
Completeness (%)^a^	99.8 (98.1)	90.2 (54.6)	98.8 (88.2)	100 (99.9)	99.9 (99.8)
Multiplicity^a^	6.7 (4.9)	5.7 (3.3)	3.5 (3.1)	28.1 (29.0)	13.0 (13.5)
Mid-slope				1.126	
dF/F				0.140	
**Refinement**					
No. of non-H atoms	11,426	11,266	28,485	21,017	21,019
R_work_/*R*_free_	0.19/0.22	0.21/0.24	0.33/0.34	0.24/0.25	0.23/0.25
Average B factors (Å^2^)	27	31	0.5	78	78
Bond length rmsd (Å)	0.0130	0.0107	0.0053	0.0087	0.0073
Bond angle rmsd (°)	1.759	1.65	1.49	1.63	1.54
Ramachandran favoured/allowed (%)	99.01/100	98/100	93.32/100	95.60/100	96/100

^a^Values in brackets are for data in the high-resolution shell.

^b^R_merg_ = Σ_*hkl*_Σ_*i*_|*I*_*i*_ – *I*_*m*_|/Σ_*hkl*_Σ_*i*_*I*_*i*_*.*

^c^R_pim_ = Σ_*hkl*_√1/*n − *1Σ_*i*=1_|*I*_*i*_ – *I*_*m*_|/Σ_*hkl*_Σ_*i*_*I*_*i*_, where *I*_*i*_ and *I*_*m*_ are the observed intensity and mean intensity of related reflections, respectively.

A structure of the SmhB pore was also determined at a resolution of 6.98 Å by molecular replacement using the AhlB pore (PDB code: 6H2F) as a search model (Supplementary Fig. 2B) (Table 1). Although the resolution of this structure was low, the electron density was clear and continuous for the protein chain, enabling a model to be built with residues truncated at Cβ. In addition, clear positive difference density could be observed in omit maps calculated with sections of the model deleted (residues 157–251 in chain A and B), indicating that the structure solution was correct (Supplementary Fig. 2C). Like the AhlB pore, the SmhB pore contained ten monomers of SmhB in two conformations (type 1 and type 2) which assemble into a ring with pseudo tenfold symmetry. As seen in AhlB, the head domain of SmhB undergoes a large scale conformational change transforming from the soluble to pore forms, with the β-tongue substructure refolding to form extended hydrophobic helices to α3 and α4 (Fig. 2B).

The *S. marcescens* Smh and the *A. hydrophila* Ahl α-PFTs have greatly varying rates of lysis of horse erythrocytes, with the AhlABC α-PFT requiring 100 times the concentration of each component to give equivalent rates of lysis as the SmhABC system^7^. As the overall structures of the soluble and pore forms of SmhB are almost identical to those of AhlB (RMSD Cα 0.64 Å and 0.46 Å, respectively), (Fig. 2C), not surprising given the high level of sequence identity (62%), we investigated if these structural similarities also conveyed functional similarities. Haemolytic assays, replacing the SmhB component of the SmhABC pore with AhlB in a 1:1:1 ratio showed that this chimeric pore had the same lytic activity as the SmhABC pore (Fig. 2C). The differences in lytic activity between the *Serratia* and *Aeromonas* tripartite α-PFTs are thus not due to the B component alone.

### Structure of soluble SmhA

To further investigate the differences in lytic activity between SmhABC and AhlABC the crystal structure of soluble SmhA was determined to 2.78 Å in space group P2_1_2_1_2_1._ This crystal form contained four closely related molecules in the asymmetric unit (rmsD Cα between molecules of 0.4–0.6 Å), with no oligomeric structures observed (Table 1) (Supplementary Fig. 2D).

**Figure 3:**
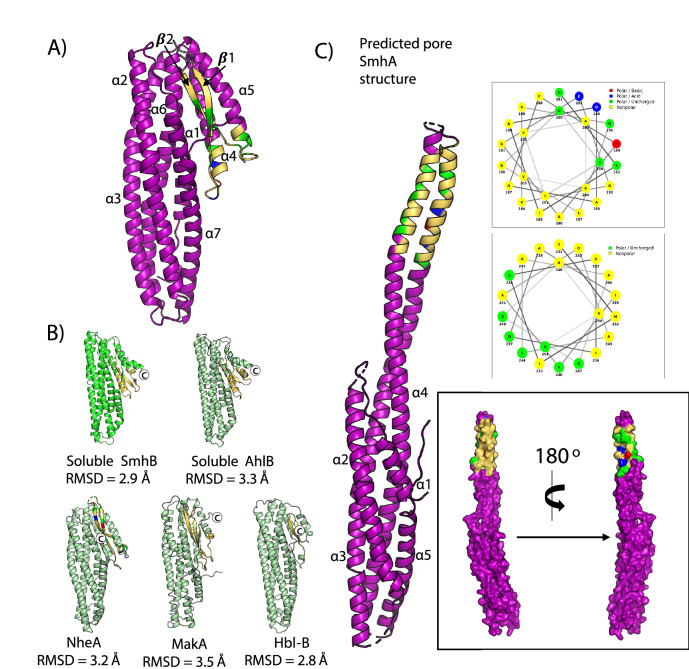
Structure of SmhA. (**A**) Crystal Structure of SmhA (purple) (PDB:7A27), with residues of the predicted head domain coloured as in helical wheels (C, top right). (**B**) rmsD values for structural alignments of soluble SmhA to soluble SmhB, AhlB, NheA, MakA, and Hbl-B. Hydrophobic residues are coloured yellow, and C-terminus is labelled. (**C**) Predicted pore structure of SmhA showing the two extended head domain helices as seen in AhlB^7^ coloured as in the plotted helical wheels (right). Surface representation of pore SmhA model (bottom right) shows how the sequence of the two extended helices create hydrophobic and hydrophilic surfaces on opposite faces of the monomer.

SmhA folds into two distinct domains, a compact five helical bundle (α1, α2, α3, α6 and α7), and an associated head domain containing two helices (α4 and α5), and the β-tongue domain (two long anti-parallel β-strands, β1 and β2) (Fig. 3A). In all monomers, there is missing density for approximately 20 residues between β1 and β2 (G208 – A229). Residues E192-V205 (β1 and loop to α3) and Y231-N250 (β2 and loop to α4) of the β-tongue domain are largely hydrophobic containing only polar uncharged and nonpolar residues, and are buried in the core of the protein, packing against α1, α3, α4, α5 (residues A272-Q283), and residues N351-E367 at the C-terminus (Fig. 3A).

Structure based sequence alignments showed that SmhA shares strong similarities with components from other tripartite systems that have a β-tongue substructure, such as SmhB (PDB code 6ZZ5, rmsD 2.9 Å), NheA (PDB code 4K1P, rmsD 3.2 Å), AhlB (PDB code 6GRJ, rmsD 3.3 Å), Hbl-B (PDB code 2NRJ, rmsD 2.8 Å), and also the *Vibrio cholera*e cytotoxin MakA (PDB code 6DFP, rmsD 3.5 Å), that is, at present, not identified as a ClyA tripartite family member (Fig. 3B). All of these structures share the same compact helical bundle tail domain and β-tongue domain. Within the tail domain, greatest variation is seen in the length of the N-terminal region. All structures have a N-terminal helix of between 22 and 28 residues, which packs against the β-tongue domain. However, in SmhA, NheA and Hbl-B, this region has an additional extended loop and short helix that extends the length of the tail domain, a feature not observed in SmhB or AhlB. In all of these structures, the C-terminus is used to shield the hydrophobic residues of the β-tongue, but the method by which the C-terminus hides the hydrophobic tail is different. MakA, NheA, and Hbl-B all use β-strands packing against the hydrophobic region of β1. AhlB and SmhB use both a short β-strand and a short helix connected by a loop, while SmhA uses only a short loop perpendicular to β1 (Fig. 3B).

It has been shown that the soluble β-tongue conformation of the ClyA family proteins rearranges into an extended pore conformation in both ClyA and AhlB^7,9^, and we have shown the same occurs in SmhB**.** As soluble SmhA, SmhB and AhlB all share very similar structures, we propose that the head domain of SmhA would also unfold into its pore form about 2 hinges in the same way as seen in SmhB and AhlB, with hinge 1 between α3 and α4, and α5 and α6, and hinge 2 between α4 and β1, and α5 and β2. This would result in the pore form of SmhA having a similar extended helical structure to that of AhlB and SmhB.

A model of this proposed pore form of SmhA was generated using a Dali^21^ structure based sequence alignment to map the residues of SmhA onto the structure of AhlB type 2 head (Supplementary Fig. 3). In this model the mixed hydrophobic/hydrophilic residues of the β-tongue head of SmhA form two extended amphipathic helices 36 Å in length at the ends of α3 and α4, (Fig. 3C), sufficient to cross the membrane, and thus with the potential to form a hydrophilic lining to the pore. In the pore conformation of SmhA, residues G208 – A229 lie between the C-terminal and N-terminal ends of the amphipathic helices α3 and α4, respectively, and would thus occur on the intracellular side of the target membrane. These residues, omitted in the soluble structure due to very weak electron density, are presumably quite flexible. This intracellular loop between α3 and α4 is present in the A components of all the Gram negative tripartite toxins, but is not found in either the Gram positive Nhe or Hbl tripartite toxins, the single component ClyA^10,22^, or bipartite YaxAB^12^ and XaxAB^13^ toxins. Residues on this loop, which do not show any pattern of conservation within the Gram-negative A component sequences, could thus interact with different intracellular components of the target cell, depending on the bacterial species the toxin is found in.

### Soluble to pore conformational change

**Figure 4:**
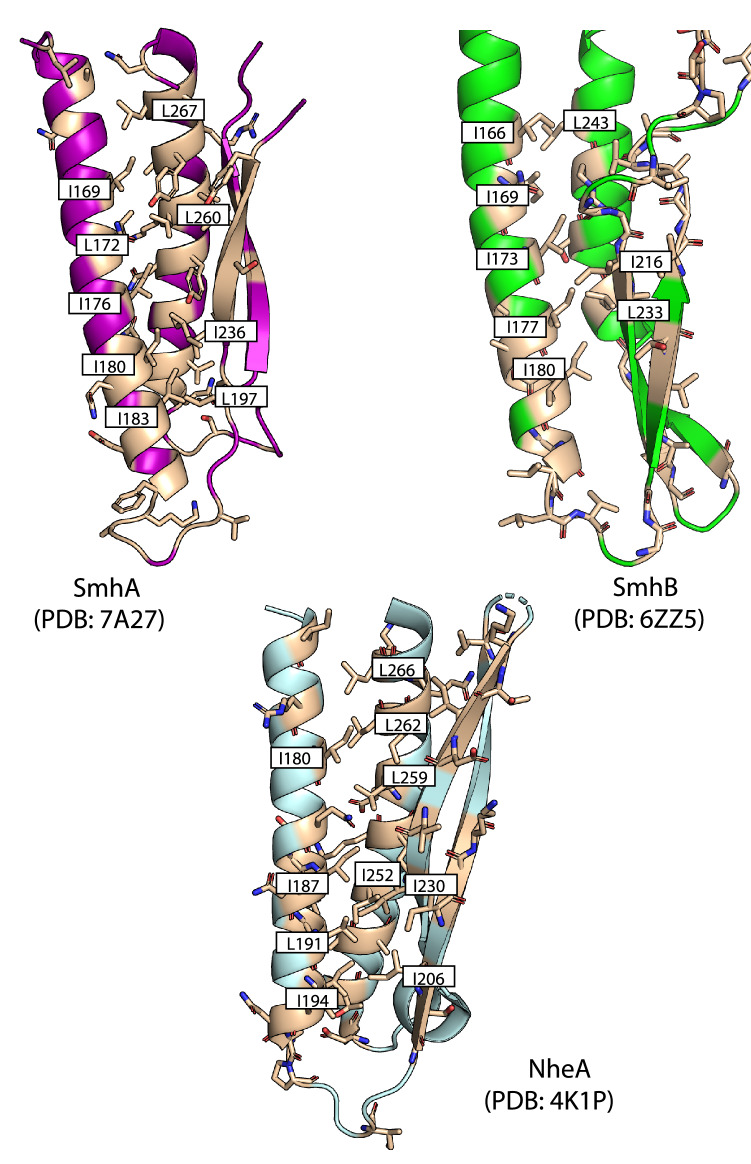
Conserved mechanisms for head domain folding. SmhA (left, purple), SmhB (right, green) and NheA (below, pale blue) adopt a leucine zipper between α4 and α5, formed from family conserved residues (light brown) to hide hydrophobic residues and stabilise the head domain.

**Figure 5:**
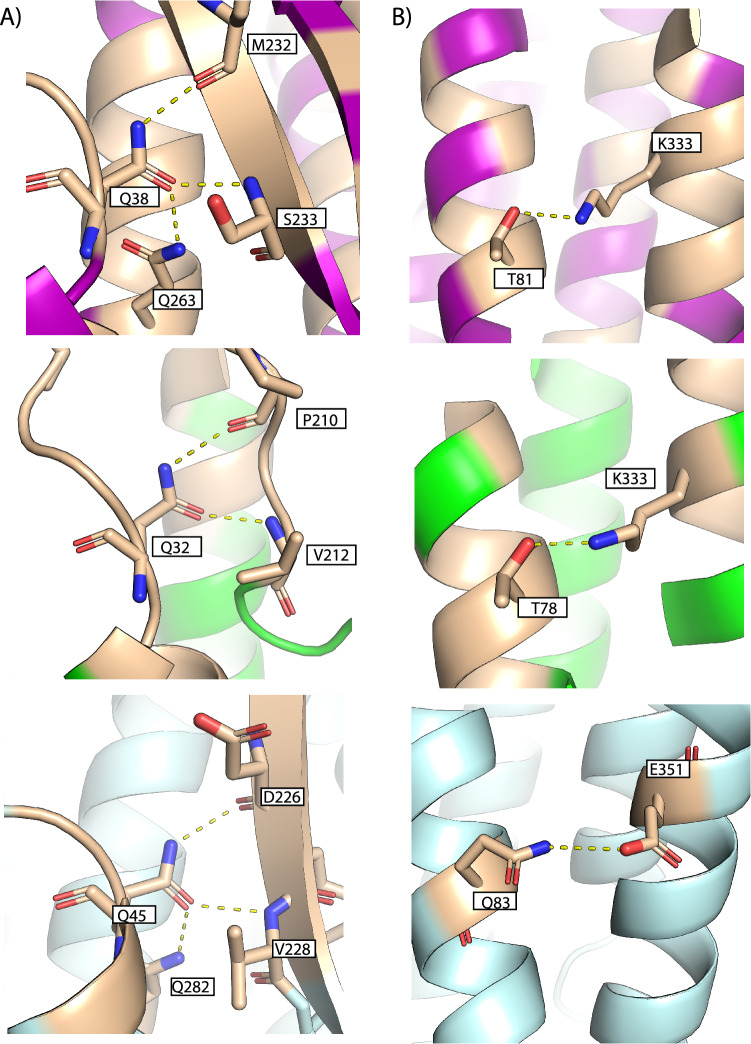
Family conserved hinge residues show conserved latch residues for head domain folding. (**A**) Hydrogen bonding between conserved glutamine residues to the main chain of the β-tongue in SmhA (top), SmhB (centre) and NheA (bottom). (**B**) Conserved Lys and Thr residues (light brown) interact between α2 and the C-terminal helix in soluble SmhA (top), and SmhB (centre), an equivalent interaction (Q83-E351) is seen in NheA (bottom).

The transformation from a soluble β-tongue containing structure to an extended helical pore structure seen in SmhB and modelled in SmhA, occurs in a similar way for *A. hydrophila* AhlB^7^. In order for this conformational change to occur, the C-terminal loop and helix α7 must move to free the β-tongue to form the two extended trans-membrane helices in the pore conformation (α3-α4), with the N-terminal helix (α1) also moving from parallel to α7 to an end to end packing, resulting in a narrower more compact tail. Sequence analysis of possible family member homologues and mapping of conserved residues onto the soluble structures of SmhA, SmhB and NheA shows that many of the conserved residues are located in the head domain and are focused on two regions. First, a leucine zipper forms between conserved Leu and Ile residues in α4 and α5 (Fig. 4), packing the hydrophobic membrane-spanning residues in the core of the domain. The second region is between the N-terminus and β-tongue, which includes a family conserved glutamine (Q38 SmhA, Q32 SmhB, Q45 NheA) on the N-terminal helix α1 that forms hydrogen bonds with the β-sheet backbone of the β-tongue (Fig. 5A). In SmhA Q38 and in NheA Q45 also interact with Q263 and Q282, respectively on α6, a residue conserved in all A component proteins (Supplementary Fig. 4). In addition, both SmhA and SmhB contain a family conserved (conserved in all but 1 sequence) lysine residue at the C-terminus (K333 in both SmhA and SmhB) that forms a hydrogen bond with a conserved hydroxyl (T81 SmhA; T78 SmhB) on helix α2 (Fig. 5B, Supplementary Fig. 4). These three motifs also occur in the structures of AhlB^7^ and NheA^23^, but in NheA a glutamate (E351) and a glutamine (Q83) hydrogen bond at the position of the Lys to Thr interaction in SmhA and SmhB (Fig. 5B). As the interactions between these conserved residues are present in the known soluble structures of all the tripartite A and B components and are disrupted during the soluble to pore transition on pore formation, it is likely that these residues play an important role in the interaction between the head and tail domains, possibly acting as latches to precipitate the conformational change in all of these β-tongue containing tripartite α-PFT proteins.

## Discussion

Studies of the ClyA family tripartite PFTs have shown a number of mechanisms by which different family members assemble their active pores. Nhe is proposed to first form BC pro-pores by binding of BC soluble complexes to the membrane before recruitment of further B to form the pro-pore, followed by the rapid addition of A and lysis^20,24,25^. Hbl first binds Hbl-B (equivalent to the SmhC component) before rapid recruitment of Hbl-L_1_ (B-component) and finally Hbl-L_2_ (A-component)^19^. Ahl primes the membrane with AhlC allowing the recruitment of AhlB and AhlA^7^. All of these three tripartite toxins also vary in their required ratio for optimum activity, with both Nhe and Hbl forming inhibitory complexes when either the C component is in excess (NheC) or the A or B component are in excess (Hbl-L_2_, Hbl-L_1_), while no inhibitory effects are seen for any of the Ahl components, with proposed ratios of 10:10:1, 1:1:10 and 1:1:1 (A:B:C) for Nhe, Hbl and Ahl respectively^7,14,18,19,24^.

**Figure 6:**
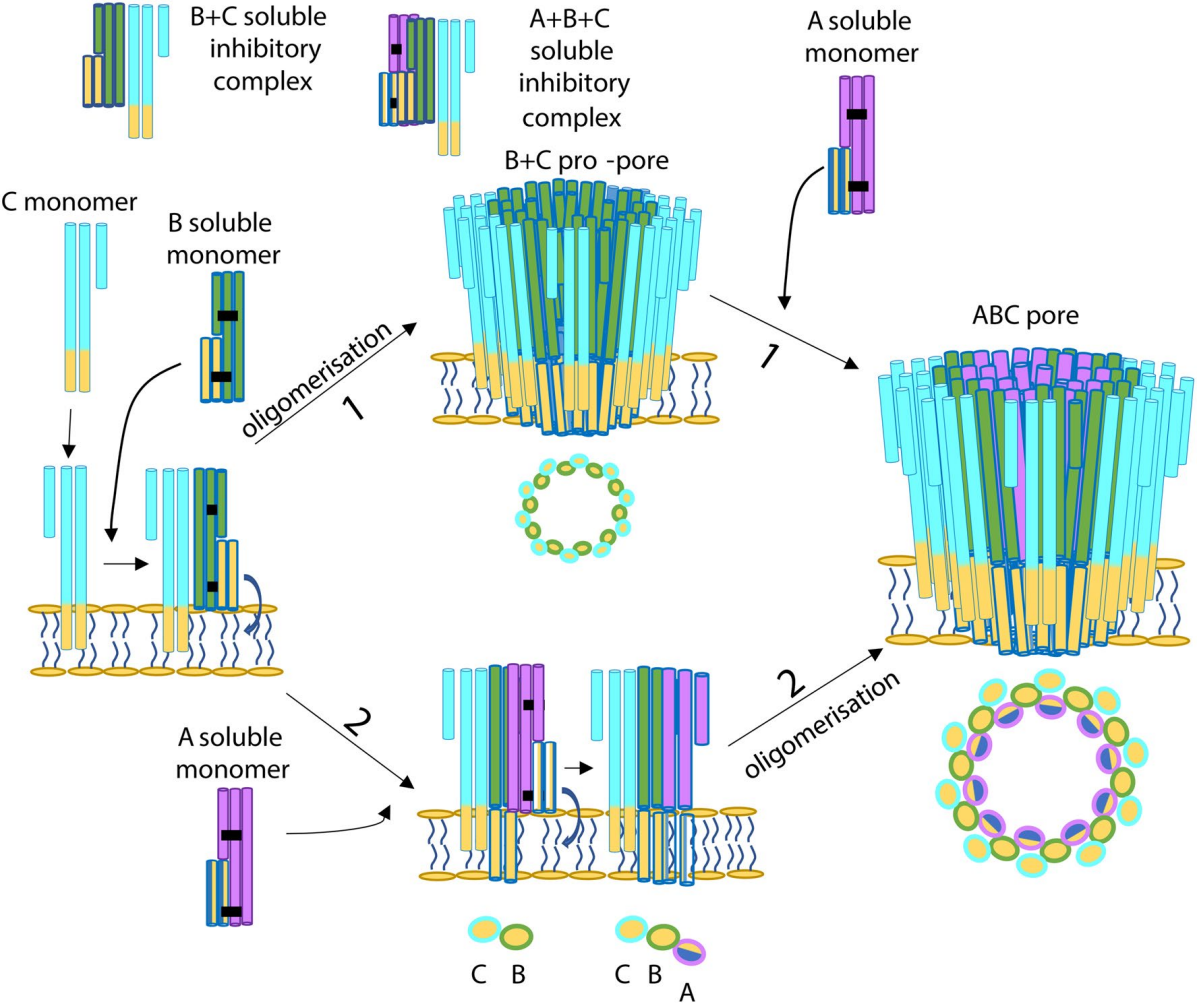
Schematic showing pore assembly for the Smh toxin. SmhC (cyan) binds to a single leaflet of the membrane using its hydrophobic head (yellow). Soluble SmhB is recruited (green) and undergoes its conformational change to pore form, with its hydrophobic head traversing the membrane. At this stage either oligomerisation occurs (top, mechanism 1) to form SmhBC pro-pores, followed by recruitment of the SmhA component (purple) to form a lytic pore. Alternatively (bottom, mechanism 2), SmhC, SmhB and SmhA assemble sequentially on the membrane prior to oligomerisation to form the lytic pore. Schematic top views of the pores are shown, with each oval representing two helices with hydrophobic (yellow) and hydrophilic (blue) surfaces indicated. Soluble inhibitory complexes can form between SmhB + SmhC and SmhA + SmhB + SmhC.

Here we have shown how *S. marcescens* contains a ClyA family tripartite PFT, Smh, which like the other tripartite family members requires all three proteins, SmhA, SmhB and SmhC to form a lytic pore. Our assays suggest that the Smh toxin employs yet another variation on the tripartite pore assembly mechanism. We have shown that the presence of both SmhB and SmhC is a minimum requirement for pore formation, with maximum lysis achieved when erythrocytes are preincubated with SmhC together with either SmhB or SmhA. Preincubation with SmhB or SmhC alone enhances lysis but not to the same extent, while pre-incubation with SmhA has no effect. This suggests that although SmhC and SmhB are both able to independently enhance lysis, in combination they are most efficient and can form pro-pores before the addition of SmhA and full lysis (Fig. 6). A similar pore assembly mechanism is also seen in the Nhe toxin^24^, but is in contrast to Ahl, where formation of AhlBC pores is an off-pathway process and inhibitory to lysis^7^. Our assays have also shown that SmhA and SmhB act to inhibit lysis when present at concentrations higher than that of SmhC, and form large inactive complexes with SmhC, a feature also shared by Hbl, but in contrast to Nhe, where NheC is inhibitory even at low concentrations^18–20^. The *A. hydrophila* AhlABC system, which is the most closely related in sequence to SmhABC, does not exhibit this inhibitory soluble complex formation, instead, the soluble AhlC component forms soluble tetramers that appear to preclude the formation of any soluble complexes with AhlA or AhlB, by occluding any binding surfaces of AhlC within the tetramer^7^. In contrast, SmhC is monomeric in solution, enabling soluble inhibitory complexes to form in the Smh toxin. Off pathway soluble complex formation is also observed in the bipartite ClyA family α-PFTs YaxAB and XaxAB^12,13^. In these toxins, both the A (equivalent to SmhC) and B components are monomeric in their soluble forms, as is also the case Hbl-B and so accessible for soluble complex formation like SmhC^12,13,26^. We propose the off pathway soluble SmhBC and SmhABC complexes act to control the efficacy of the Smh toxin. Although there is still some ambiguity in the exact order of membrane association for the Smh toxin, our TEM and haemolytic assays show that SmhBC pro-pores are definitely able to assemble, with SmhA interacting with the SmhBC pro-pore to destroy the cell. However, our data are also consistent with the Smh components binding sequentially at the membrane to form a fully lytic pore, as seen with Hbl^19^ (Fig. 6).

**Figure 7:**
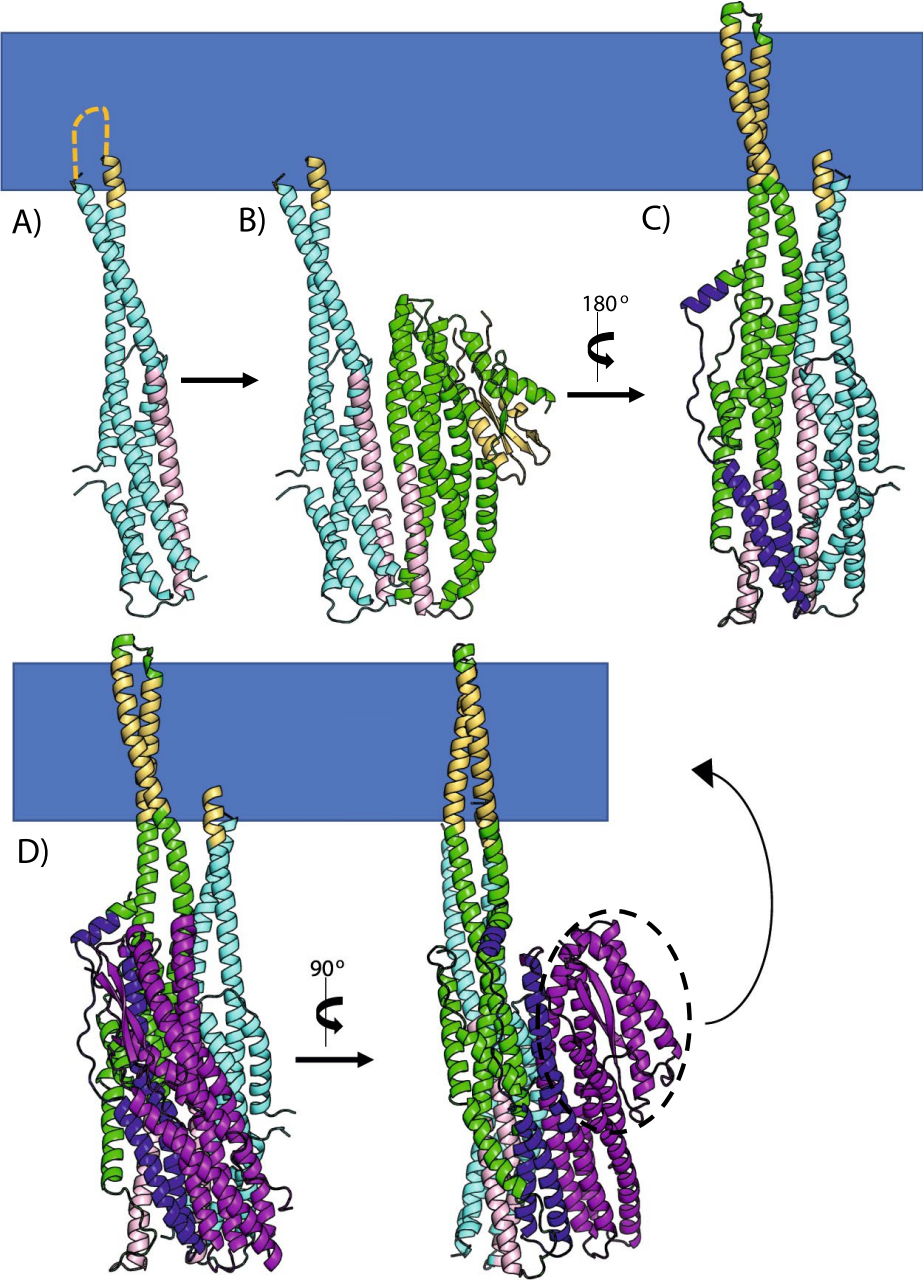
Model of SmhABC tripartite α-PFT pore assembly. (**A**) The C component (cyan) inserts its hydrophobic head (yellow) through a single leaflet of the membrane. (**B**) Soluble B (green) binds to C with the N-terminal residues of α3 (G97- R125) in B interacting with the C-terminal residues of α3 on C (regions of both coloured pink). (**C**) The B component then undergoes a large conformational change, inserting two hydrophobic helices through the membrane. (**D**) Finally, the A component (purple) binds to the B component with the region Q58-T72 on A (blue) interacting with E294-L313 on B (blue). The head region of A (circled in dashed lines) is predicted to undergo a conformational change to extended helices, which insert through the membrane. All complexes modelled using the HADDOCK2.2 web server^29^.

We have identified two potential latches in the soluble tripartite β-tongue containing components, and the residues involved are located in surface patches of conserved residues in both SmhA and SmhB (Supplementary Fig. 5). Monoclonal antibody binding studies that interfere with interactions between the three components of the *B. cereus* Nhe α-PFT^24,27,28^ have identified the binding sites between NheA (Mab site 2G11) to NheB (Mab site 1E11) and NheB (Mab site 2B11) to NheC (Supplementary Fig. 6). Mapping of these binding sites onto the SmhA and SmhB structures, (Supplementary Fig. 6), show that SmhC would interact with SmhB at the patches of conserved residues on SmhB containing both latch 1 and latch 2. Likewise, the interaction of SmhB with SmhA would also involve the latch motifs on SmhA. It thus seems likely that the β-tongue containing components of the tripartite ClyA family toxins share a conserved mechanism of transformation from soluble to pore form. The B and C components interact at a binding interface located around residues 95–123 of SmhB, this could break latches 1 and 2 on the B component, releasing the β-tongue, C-terminus, and N-terminus to initiate the transformation into the B component pore conformation (Fig. 7). The A component then binds to B at an interface around residues 289–308 in SmhB and 57–71 in SmhA, similarly breaking the two latches on the A component, and allowing its conformational change from soluble to pore form (Fig. 7). The full conformational change of the β-tongue to the pore forms of both A and B components requires a membrane, otherwise off pathway soluble complexes occur^12,18,20^. The release of the C-terminus from the β-tongue agrees with the observation that the start of the conformational transition in NheA occurs at the C-terminus^24^. This is further evidenced in the structure of a second crystal form of SmhB (Table 1), where comparison of the B-factors for the two chains in the asymmetric unit revealed high flexibility at residues 85–124 and residues 280–320 (Supplementary Fig. 7), corresponding to the proposed binding sites to SmhC and SmhA, indicating that movement in these binding regions can occur to facilitate complex formation.

**Figure 8:**
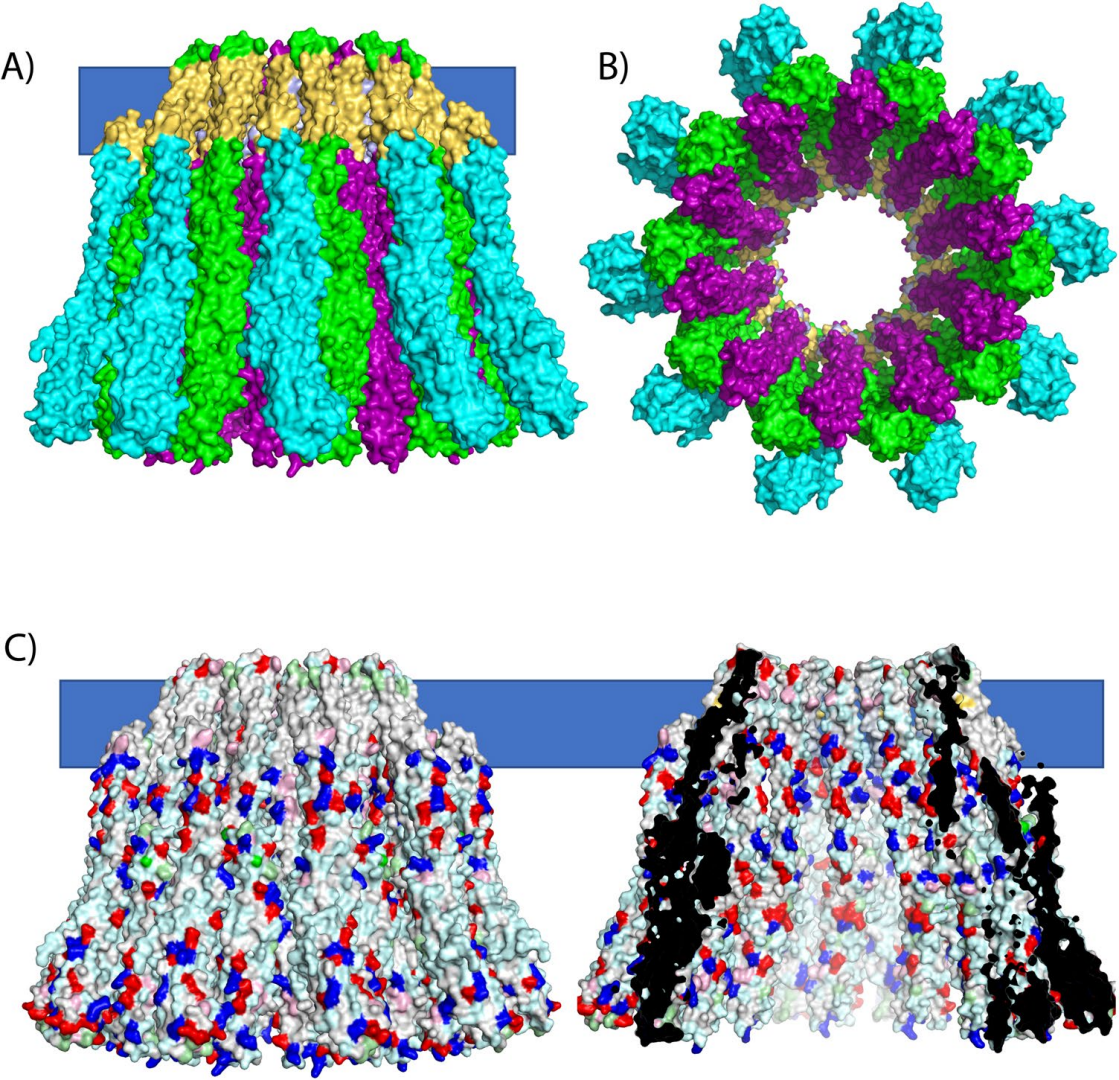
Model of the complete tripartite α-PFT ABC pore. (**A**) Based on docking predictions the A component (purple) fits between neighbouring B components (green) on the inside surface of the pore, surrounded by C components (cyan), highlighting the membrane (blue bar) and hydrophobic surfaces (yellow). (**B**) Top view of pore. (**C**) Surface rendered full and cutaway side views of the pore model generated in Pymol^46^, showing the hydrophilic lining to the pore with residues coloured hydrophobic (white), polar (pale blue), positive (blue) and negative (red).

These predicted binding sites between the A, B and C components, the structures of SmhA, SmhB and AhlC (as a surrogate for SmhC), and structure based sequence alignments were used to construct models of AhlC bound to soluble SmhB; AhlC bound to SmhB type 2 pore conformation and the AhlC: SmhB complex bound to soluble SmhA with the HADDOCK server^29^ (Fig. 7). Ten of these SmhA, SmhB, SmhC units were subsequently assembled into a model of the complete tripartite pore with C10 symmetry by rotation of increments of 36° around the central axis of the SmhB pore (Fig. 8). This model of the SmhABC pore is consistent with that proposed for AhlABC^7^ and also by extension, given the levels of sequence and structural similarity, for other tripartite ClyA family α-PFTs^7^. This pore structure has an inner ring of alternating membrane-spanning hydrophobic B and amphipathic A components forming the hydrophilic pore, surrounded by the single leaflet anchoring C components. This general pore architecture is also seen in the greater ClyA family, but with the individual roles of the three tripartite PFT proteins of a single leaflet anchoring function, a membrane-spanning function and a hydrophilic pore lumen carried on two proteins for the bipartite YaxAB/XaxAB PFTs^12,13^ and on a single protein for the prototypical ClyA toxin^9,10^. Within the tripartite α-PFT family members, both the structures of the A and B components and the mechanism for transformation from soluble to pore forms of these components are well conserved. Nevertheless, the involvement of three proteins in pore formation has enabled differences in both the assembly and in the regulation of each tripartite PFT system, helping these bacterial species to thrive in their different environmental niches.

## Materials and methods

### Protein cloning and purification

The ORFs for SmhA, SmhB, and SmhC from *S. marcescens* MSU-97 were synthesised and cloned into pET21a expression vectors by GenScript Biotech Corporation, so as to contain a C-terminal 6-His tag. Each protein was expressed in *E.coli* BL21 DE3 expression cell line (NEB). Cultures were grown in LB media at 37 °C until an OD_600_ 0.6 was reached, protein expression was then induced using 1 mM isopropyl β-D-1-thiogalactopyranoside (IPTG). For all three proteins expression was carried out at 16 °C overnight. SmhA and SmhB were purified using the same protocol. The cell pellet was resuspended in lysis buffer (50 mM Tris pH8) and sonicated (3 × 20 s burst at 16,000 nm λ), insoluble material was removed by centrifugation at 40,000 g for 15 min. Soluble protein in binding buffer (50 mM Tris pH8, 0.5 M NaCl) was applied to a 5 ml Nickel Hi-trap column (GE Healthcare) and protein was eluted using a gradient of 0–1 M imidazole in binding buffer. Protein was further purified by size exclusion chromatography on a Superdex 200 pg column (GE Healthcare) pre-equilibrated with 50 mM Tris pH8, 0.5 M NaCl. The SmhC cell pellet was sonicated and centrifuged as described for SmhA and SmhB. As the protein did not bind to the Ni column, an ammonium sulphate cut was carried out on soluble SmhC to a final ammonium sulphate concentration of 0.5–1.5 M. After 10 min precipitated protein was pelleted by centrifugation at 70,000 g for 5 min at 4 °C, the pellet was resuspended in 50 mM Tris pH8. Soluble protein was applied to a 10 ml (2 × 5 ml) HiTrap DEAE Sepharose Fast Flow column (GE Healthcare) pre-equilibrated in 50 mM Tris pH 8 and eluted on a gradient of 0–0.3 M NaCl in 50 mM Tris pH8, 1 M NaCl. Selenomethionine SmhA protein was expressed in the same way as the S-met SmhA, as detailed previously^30^.

### Generation of homologue sequence alignments

Homologues were identified using the BlastP server^31^ and all results with an E < 0.01 were assessed to determine if they were part of a tripartite toxin operon. All homologues identified as part of a tripartite PFT were then used in subsequent sequence alignments. Sequence alignments of homologues of SmhA and SmhB were generated using Tcoffee^32^.

### Haemolytic assays

The lytic activity against horse erythrocytes of SmhA, SmhB, and SmhC was determined by measuring the release of haem from lysed cells as described by Rowe and Welch^33^. A 0.5% v/v suspension of horse erythrocytes (Thermo scientific) was prepared by repeated washing of cells and resuspension in 10 mM PBS pH 7.4. Varying concentrations of each SmhABC protein were incubated with 0.1 ml erythrocyte suspension on a blood wheel at 37 °C for 1 h, or cells were preincubated with various components for one hour followed by addition of the remaining components followed by a further 1 h incubation. Erythrocyte mixtures were centrifuged at 1500 g for 5 min, and supernatant removed for photometric analysis at 542 nm. A positive control of erythrocytes lysed with ddH_2_O and a negative control with no protein were used to normalise the data for 0 and 100% lysis. All assays were carried out in triplicate (n = 3), and analysed for significance using an unpaired T-test .

### Analysis of soluble complexes of SmhABC

To determine whether individual components of Smh could bind to each other in the soluble conformation, 40 $\mathrm{\mu M}$ of each protein in 10 mM PBS pH 7.4 were mixed in various combinations and incubated for 45 min at 37 °C and then applied to a Superdex 200 increase gel-filtration (GE Healthcare) column pre-equilibrated with PBS. Fractions were collected and analysed by SDS-PAGE.

### SmhA crystallisation and structure determination

Purified SmhA was concentrated to 7 mg/ml in a Vivaspin 30 KDa MWCO concentrator (Sartorius), and then buffer exchanged into 50 mM Tris pH8, 10 mM NaCl. Crystals were grown by sitting drop vapour diffusion in 96-well plates. Se-methionine crystals grew in 0.1 M MES pH 6.5, 0.16 M CaCl_2_ and 20% PEG 6000 (7 °C, 200 nl:200 nl drop), while S-methionine crystals grew in 0.2 M potassium nitrate, 20% PEG 3350 (7 °C, 200 nl:200 nl drop). Crystals were cryo-protected in mother-liquor containing an additional 20% (v/v) ethylene glycol before freezing in liquid nitrogen.

X-ray diffraction data from a single Se-methionine SmhA crystal were collected on beamline i03 of the Diamond Light Source (DLS) at wavelength 0.9792 Å. Images were integrated and scaled using the Xia2 Dials pipeline^34,35^ into space group P4_2_ at a resolution of 2.98 Å (Table 1). Initial phases were obtained by SAD, with the heavy atom sites and initial electron density map and model calculated using the CRANK2 pipeline^36^. The model was optimised and completed in Coot^37^. This crystal form 1 (PDB code 7A26) contained eight independent molecules in the asymmetric unit and the structure of a single chain was used as a molecular replacement model to determine the structure for a second higher resolution S-methionine data set (2.57 Å), in space group P2_1_2_1_2_1_. This second data set was collected on beamline i04-1 of the DLS at wavelength 0.9159 Å and images were processed using Xia2 3dii (Table 1). Rebuilding and refinement were carried out using COOT and REFMAC^38^, to give a final model with R and Rfree of 0.23 and 0.25, respectively (PDB code 7A27). Residues 210–228 in chain A, 209–228 in chain B, 209–225 in chain C and 210–225 in chain D were omitted from the final model due to poor density.

### SmhB crystallisation and structure determination

Purified SmhB was concentrated to 14 mg/ml in a Vivaspin 30 KDa MWCO concentrator (Sartorius) and then buffer exchanged into 50 mM Tris pH8, 10 mM NaCl. All crystals were grown by sitting drop vapour diffusion in 96-well plates. Crystals grew in 0.17 M ammonium sulphate, 25.5% PEG4000 (16 °C, 100 nl:100 nl drop) and were cryo-protected in mother-liquor containing an additional 20% (v/v) ethylene glycol before freezing in liquid nitrogen. X-ray diffraction data from a single SmhB crystal was collected on beamline i04-1 of the DLS at wavelength 0.9159 Å. Images were integrated and scaled using the Xia2 3dii^35^ pipeline into space group P2_1_2_1_2_1_ at a resolution of 1.84 Å (Table 1). The structure was determined by molecular replacement with PhaserMR^39^ using the soluble conformation of AhlB (PDB: 6GRK) as a search model. Rounds of model building and refinement were carried out using COOT^37^ and REFMAC^38^ to give a final model with an R and Rfree of 0.19 and 0.22, respectively (PDB code 6ZZ5). Residues 206–207 in chain A and 206–208 in chain B were omitted from the final model due to poor density.

A second crystal form of SmhB was grown in 0.22 M Magnesium chloride hexahydrate, 0.1 M Na acetate pH 5.15, 26% (w/v) PEG 6000 (16 °C, 100 nl:100 nl). Data was collected on beamline i04-1 of the DLS. Images were integrated and scaled using AutoPROC + STARANISO^40–44^ into space group P2_1_ at a resolution of 1.86 Å. The structure was determined by molecular replacement with PhaserMR^39^ using a single chain of the Form 1 SmhB structure as a search model. Rounds of model building and refinement were carried out using COOT^37^ and REFMAC (39) to give a final model with an R and Rfree of 0.21 and 0.25 respectively (PDB code 6ZZH). Residues 205–206 in chain A and B were omitted from the final model due to poor density.

The pore structure of SmhB was crystallised in 0.2 M Calcium chloride, 40% MPD (16 °C, 100 nl:100 nl). Data were collected from on beamline i24 of the DLS at wavelength 0.9795 Å. Images were integrated and scaled using Dials^34^ into space group C2 at a resolution of 6.98 Å. The structure was determined by molecular replacement with PhaserMR (40) using the AhlB pore conformation (PDB: 6GRJ) as a search model. Rounds of model building and refinement were carried out using COOT (38) and REFMAC^38^ to give a final model with an R and Rfree of 0.33 and 0.33 respectively (PDB code 7AOG). The final model was constructed from poly-alanine as density for side chains could not be resolved at this resolution.

Structure alignments in this paper were done using Dali^21^.

### Modelling NheB and C, and docking of A, B, C complexes

Homology models of NheB and NheC were generated using Phyre2^45^ and the structure of Hbl-B (PDB: 2NRJ) as the template. Models of potential complexes between the components in the tripartite α-PFT family were generated using structures of individual components from the Smh and Ahl systems, predicted binding regions determined from antibody binding studies on the Nhe system and the HADDOCK2.2^29^ server. Multiple runs of Haddock were completed and only consistent solutions were used in subsequent modelling. In this way models of complex structures between AhlC (PDB:6H2E) and SmhB (PDB:6ZZ5) (predicted docking regions residue 97–125 in SmhB, 79–118 in AhlC chain Q); AhlC (PDB:6H2E) and AhlB T2 (PDB: 6H2F) (residues 97–125, 176–184 and 227–234 AhlB T2 , 79–118 and 153–171 in AhlC chain Q); and AhlBT2 (PDB: 6H2F) and SmhA (PDB: 7A27) (residues 289–319 in AhlBT2, 57–71 in SmhA) were produced.

### Electron microscopy

Lytic assay samples were visualised using negative stain TEM. 5 µl of each respective sample was pipetted onto a glow discharged carbon-coated grid (copper 300 mesh), then stained with 1% (w/v) uranyl formate and air dried. Electron micrographs were collected with a Philips CM100 100 kV transmission electron microscope, equipped with a Gatan 1 K CCD camera. Micrographs were collected with a pixel to nm ratio of 0.72 pixels per nm.

### Generation of figures

All protein cartoon and surface rendering used in the figures were generated using Pymol^46^.

## Supplementary Information

Supplementary Information

## Data Availability

Data supporting the findings of this manuscript are available from the corresponding author upon reasonable request. Atomic coordinates for SmhA crystal form1 (PDB code 7A26), SmhA Form 2 (PDB code 7A27), soluble SmhB form1 (PDB code 6ZZ5), soluble SmhB form2 (PDB code 6ZZH), SmhB pore (PDB code 7AOG), have been deposited in the RCSB Protein Data Bank.
