# Supplementary material for: Characterisation of a tripartite α-pore forming toxin from Serratia marcescens
Source: Sci Rep. 2021 Mar 19;11:6447. doi: 10.1038/s41598-021-85726-0 (PMC7979752; doi:10.1038/s41598-021-85726-0)
Supplement: Supplementary file 1 — Supplementary Information [file 41598_2021_85726_MOESM1_ESM.pdf]

Characterisation of a tripartite α-pore forming toxin from *Serratia marcescens.*

Alicia M. Churchill-Angus[1], Thomas H B Schofield[1,2], Thomas R. Marlow[1], Svetlana E. Sedelnikova[1], Jason S. Wilson[1], John B. Rafferty[1], and Patrick J. Baker[1*]

Supplementary information

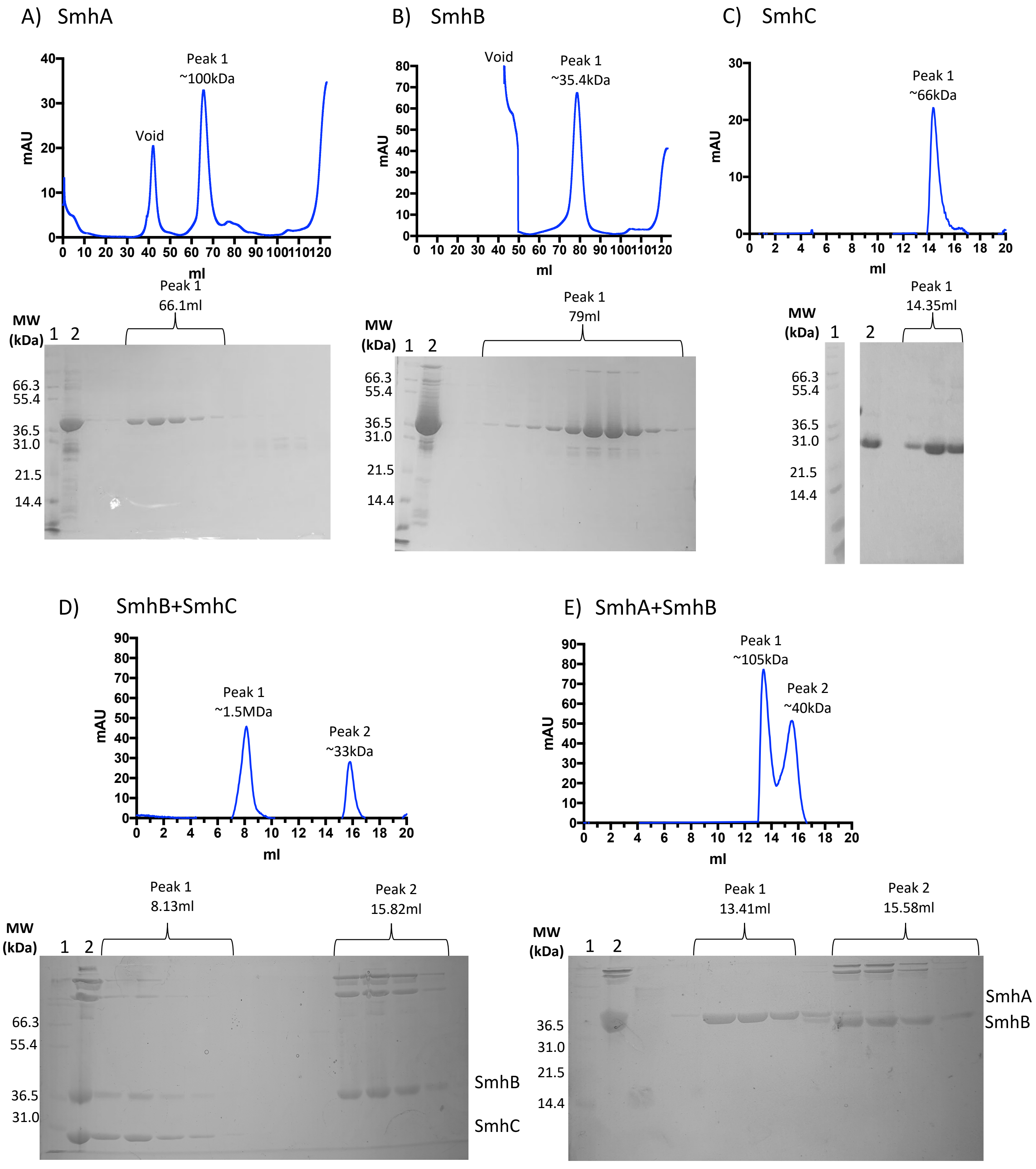
A) SmhA
mAU
ml
Void
Peak 1
~100kDa
Peak 1
66.1ml
MW
(kDa)
1
2
66.3
55.4
36.5
31.0
21.5
14.4
B) SmhB
mAU
ml
Void
Peak 1
~35.4kDa
Peak 1
79ml
MW
(kDa)
1
2
66.3
55.4
36.5
31.0
21.5
14.4
C) SmhC
mAU
ml
Peak 1
~66kDa
Peak 1
14.35ml
MW
(kDa)
1
2
66.3
55.4
36.5
31.0
21.5
14.4
D) SmhB+SmhC
mAU
ml
Peak 1
~1.5MDa
Peak 2
~33kDa
Peak 1
8.13ml
Peak 2
15.82ml
MW
(kDa)
1
2
66.3
55.4
36.5
31.0
SmhB
SmhC
E) SmhA+SmhB
mAU
ml
Peak 1
~105kDa
Peak 2
~40kDa
Peak 1
13.41ml
Peak 2
15.58ml
MW
(kDa)
1
2
36.5
31.0
21.5
14.4
SmhA
SmhB

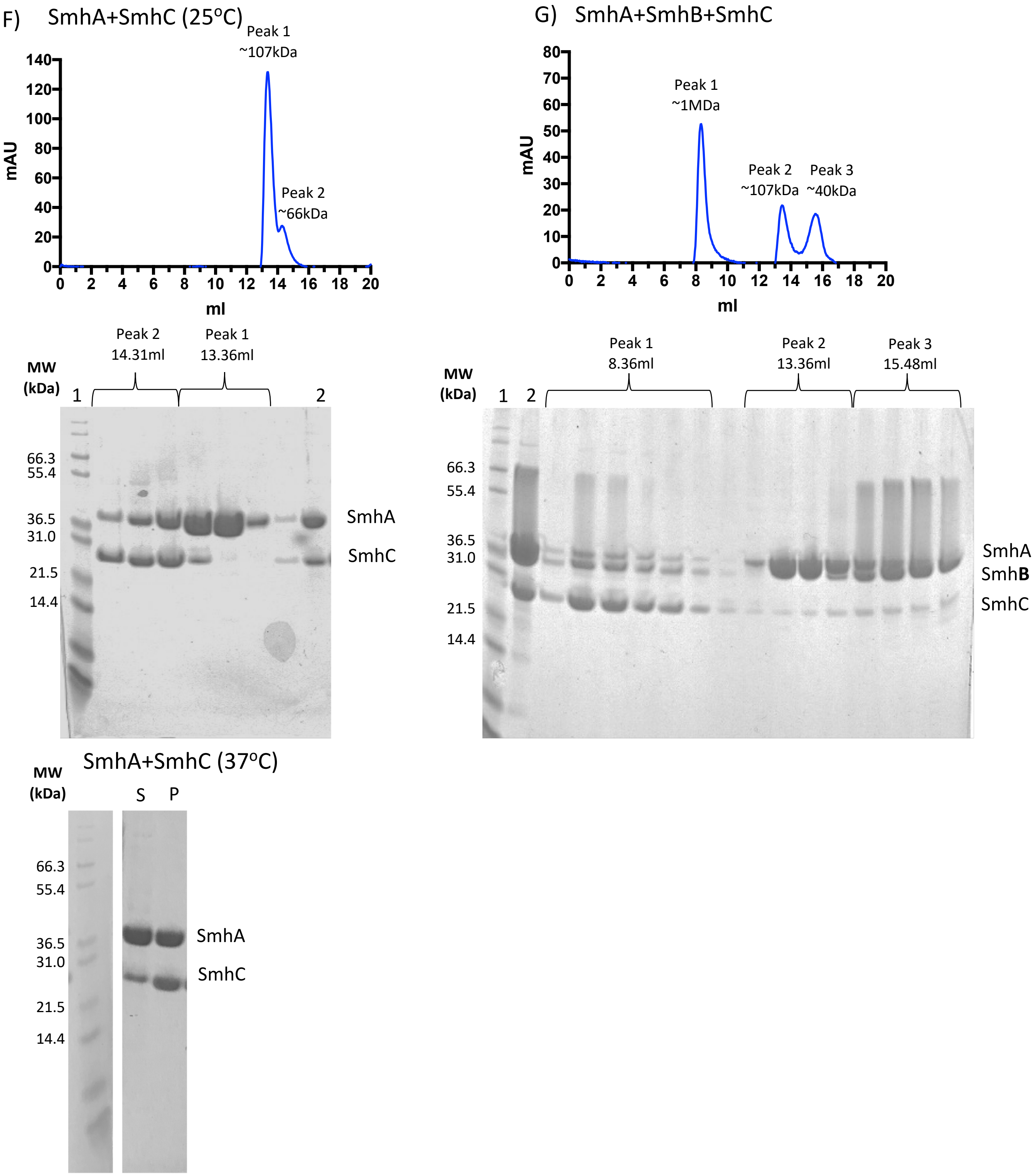

Supplementary Figure 1. Analysis of SmhA, SmhB and SmhC by size exclusion chromatography. Gel filtration chromatograms with SDS-PAGE gels below showing the content of the labelled peaks, Lane 1 contains the MW ladder, Lane 2 contains the gel filtration load. A) SmhA purification. Superdex 200pg B) SmhB purification. C) SmhC purification. Lane 1, 2 and peak1 are cropped images from the same gel. D) A 1:1 ratio of SmhB + SmhC elutes as 2 peaks, one high molecular weight peak containing both SmhB and SmhC and a second peak low mw peak containing only SmhB. E) A 1:1 ratio of SmhA + SmhB elutes as 2 separate peaks one containing SmhA and the other SmhB. F) A 1:1 ratio of SmhA + SmhC , peak 1 containing SmhA and peak 2 containing SmhC. SDS-PAGE of resulting supernatant (S) and pellet (P) after incubation of SmhA and SmhB at 37°C for 1 hr (below), showing that most of SmhC precipitates with SmhA. Ladder, lane S and P are cropped images from the same gel. G) A 1:1:1 ratio SmhA + SmhB +SmhC which elutes as 3 separate peaks, peak 1 – high molecular weight species containing SmhA, SmhB and SmhC. Peak 2 and 3 contain the soluble SmhA, SmhB and SmhC components. A) and B) purified using Superdex 200pg, C)-G) purified using Superdex 200 increase.

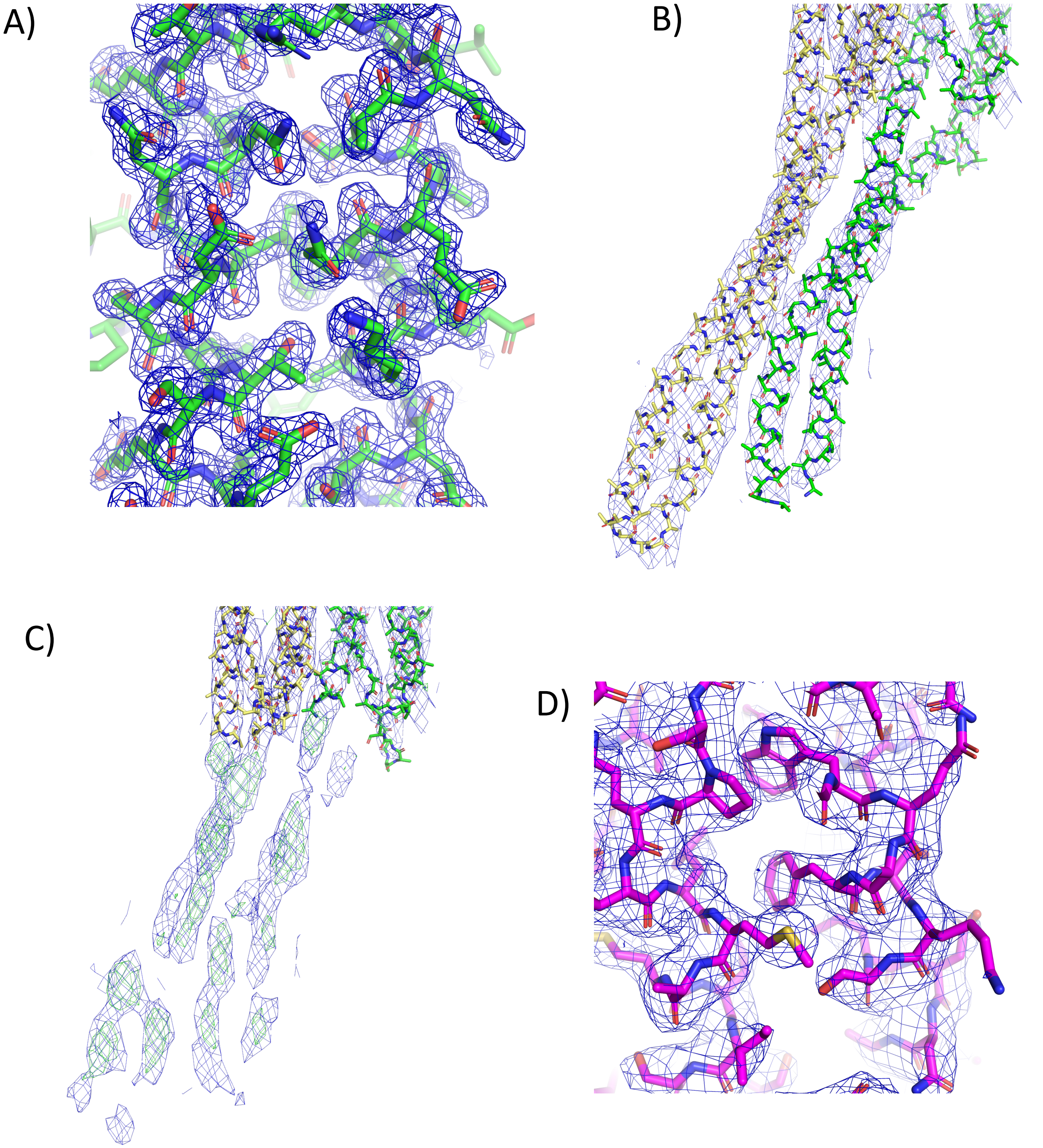

Supplementary Figure 2. Electron density maps. A) 1.8 Å resolution 2Fo-Fc map contoured at 1.0σ for soluble SmhB residues N122-D111 and E293-N282 B) 6.98 Å resolution 2Fo-Fc map contoured at 1.0σ (blue mesh) for SmhB pore conformation chain A (yellow) and B (green) . C) 2Fo-Fc map contoured at 1.0σ (blue mesh) and positive difference map contoured at 2.99σ (green mesh) from an omit map after refinement deleting the head region of chains A and B from the SmhB pore. Positive density returns for both head domains. D) 2.6 Å resolution 2Fo-Fc map contoured at 1.0σ for SmhA residues W342-N351 and F345 and S10-S20.

```
DSSP   lhhhllllLLLHHHHHHHHHHHHHHHHHHHHHHHHLLLLLLLLL--LHHHHHHHHHHHHH
SmhA   nltsidlsPQTLMAMHISISSQALLNQSYSNLLLSQQLLTSQSM--DPGLTVKIKAYQNQ   58
ident                     |||||  | | |    |                 |
SmhB   ------ipTLYMNDGMNAQSSQALHIQTYCNSVRQQIPVDFGRFpnLRESERQINTGLGA   54
DSSP   ------lhHHHHHHHHHHHHHHHHHHHHHHHHHHLLLLLLLLLLhhHHHHHHHHHHHHHH

DSSP   HHHHHHHHHHLHHHHHHHHHHHHHHHHHHHHHHH-HHLLLllllHHHHHHHHHHHHHHHH
SmhA   LRQQAQVFKQNTVAELIGLYTKASNFAALVNAVN-ALYSTedpqVSQKGAEMVAALSDVA  117
ident   || |           |   |         |       |                 || |
SmhB   ARQHAEHYLKDIQPLIIRNVTNIQDYFETQNLIStVMPSG---aTKEQWLSALGMVSDKA  111
DSSP   HHHHHHHHHHHLHHHHHHHHHHHHHHHHHHHHHHhHLLLL---lLHHHHHHHHHHHHHHH

DSSP   HHHHHHHHHHHHHHHHHHHHHHHHHHHHHHHHHHHHLLLLhhHHHHHHHHHHHHHHHHHH
SmhA   QHYQAAAQAVHTQLQAKREMLEPLMGNFLNVIDAIEQGLNaeAKQQAQTIAELNEAIAKN  177
ident    ||                |     |            |                |
SmhB   KEYQEVSANTRRTIGSLNDKLIIDSNNYQLIVVNLNNVVN-gNNGVLEQLNRDIDGINAA  170
DSSP   HHHHHHHHHHHHHHHHHHHHHHHHHHHHHHHHHHHHHHLL-lLHHHHHHHHHHHHHHHHH

DSSP   HHHHHHHHH-HHHLL---LLLLEEEEEEEeellllllLEEEEE--EEELL----lllLLL
SmhA   IQSIADAGF-KAGEG---VVQLGQSIVAAvplgpadqASYMIS--GIQAI----sagASA  227
ident  |                         ||         ||      ||
SmhB   IDGAIAGIVvGGLLViggAIVTAIGAVAG-----lvtASTPVVmgGIAMMtagaggvIGG  225
DSSP   HHHHHHHHHhHHLLLlllEEEEEELLLLL-----lllLLLLLLllLEEEEeelllllEEL

DSSP   LHHHHHHHHHHHHHHHHhHHHHhllLHHHHHHHHHHHHHHHHHHHHHHHHHHHLLHHHHH
SmhA   AQQAVNELKANYAKLAVaYRALataNALLSVAKSVQAQAQLFVDTYVLTEQRMALLPTEW  287
ident  |      | |           |   |    ||                           |
SmhB   AIVLDKSLSAREKLYRD-RSQL---NSEVLVASQIGSGYRGLQTQAQSAVTAATQMNNAW  281
DSSP   HHHHHHHHHHHHHHHHL-LLLL---LHHHHHHHHHHHHHHHHHHHHHHHHHHHHHHHHHH

DSSP   HHHHHHHHHHHHHHHHLllhhHHHHHHHHHHHHH-HHHHHHHHHHHHHHHHHHHLlLLLL
SmhA   GKVAEAYLTAAPIINQAasaaEIKQAKQIISLNA-EKWQLFSKSIDNAKANYAGNnILPE  346
ident          |                  |                    |   ||     |
SmhB   DSLTSELETLNANLRKG--iiDDSFLRQLFLTASqTSVTKVLDGTKIIKQQMAGV-VVRE  338
DSSP   HHHHHHHHHHHHHHHLL--llLHHHHHHHHHHHHhHLHHHHHHHHHHHHHHHHLL-EELL

DSSP   LL---------------l
SmhA   VL---------------e  349
ident  |
SmhB   VPanqsiadfvkrlaale  356
DSSP   LLllllhhhhhhhhhhhl
```

Supplementary Figure 3. Structure based sequence alignment of SmhA and SmhlB, helices shown as rectangles, strands as arrows (H-helix, L- loop, E-strand), prepared using Dali[1] .

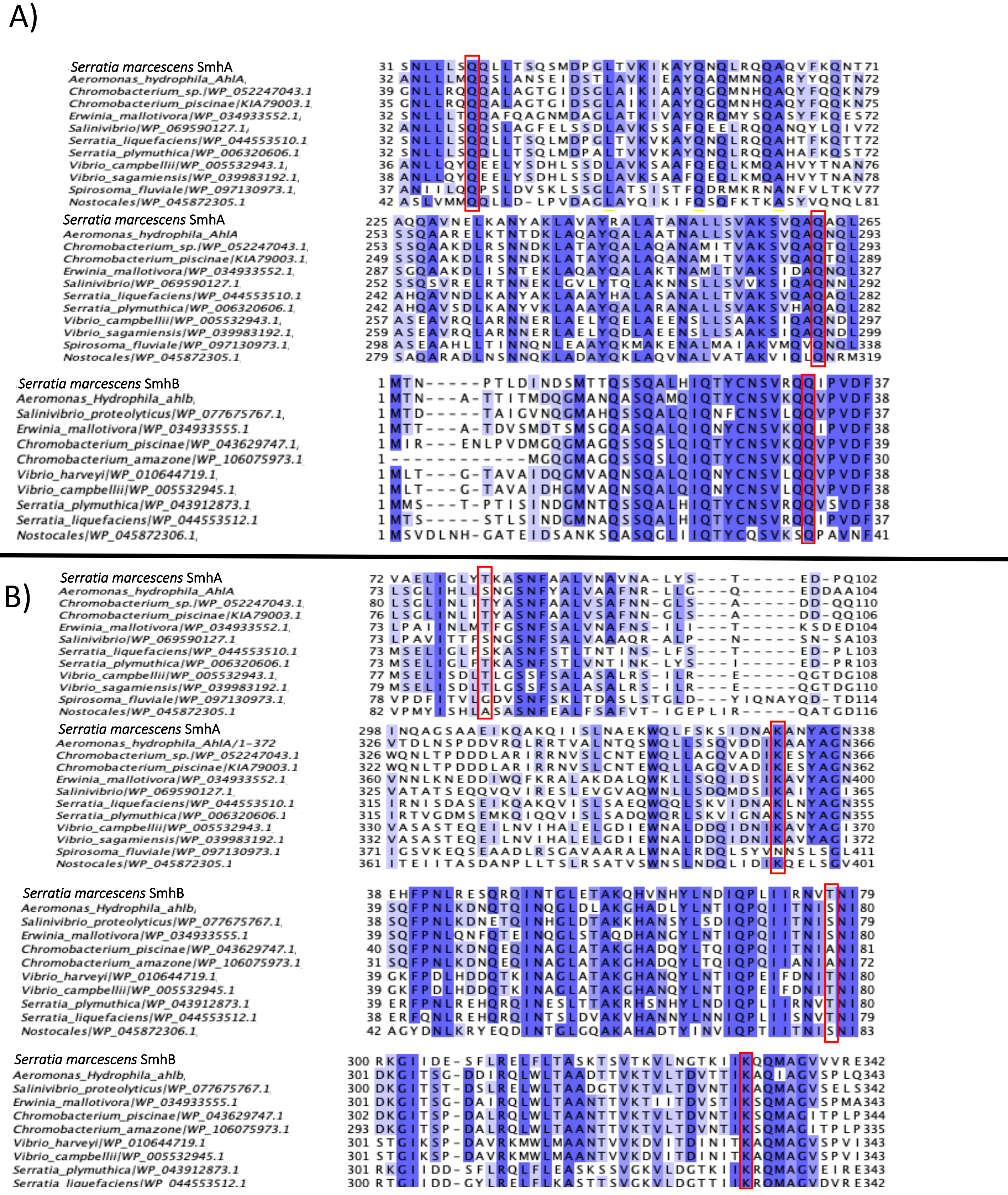

Supplementary Figure 4. Sequence alignments of SmhA and SmhB with A and B components from other species, highlighting (red boxes) residues involved in hydrogen bonding networks that are broken on the soluble to pore transition and with family conserved residues in blue. A) Q38 and Q263 in SmhA and Q32 in SmhB. B) T81 and K333 Lys in SmhA and T78 and K333 SmhB.

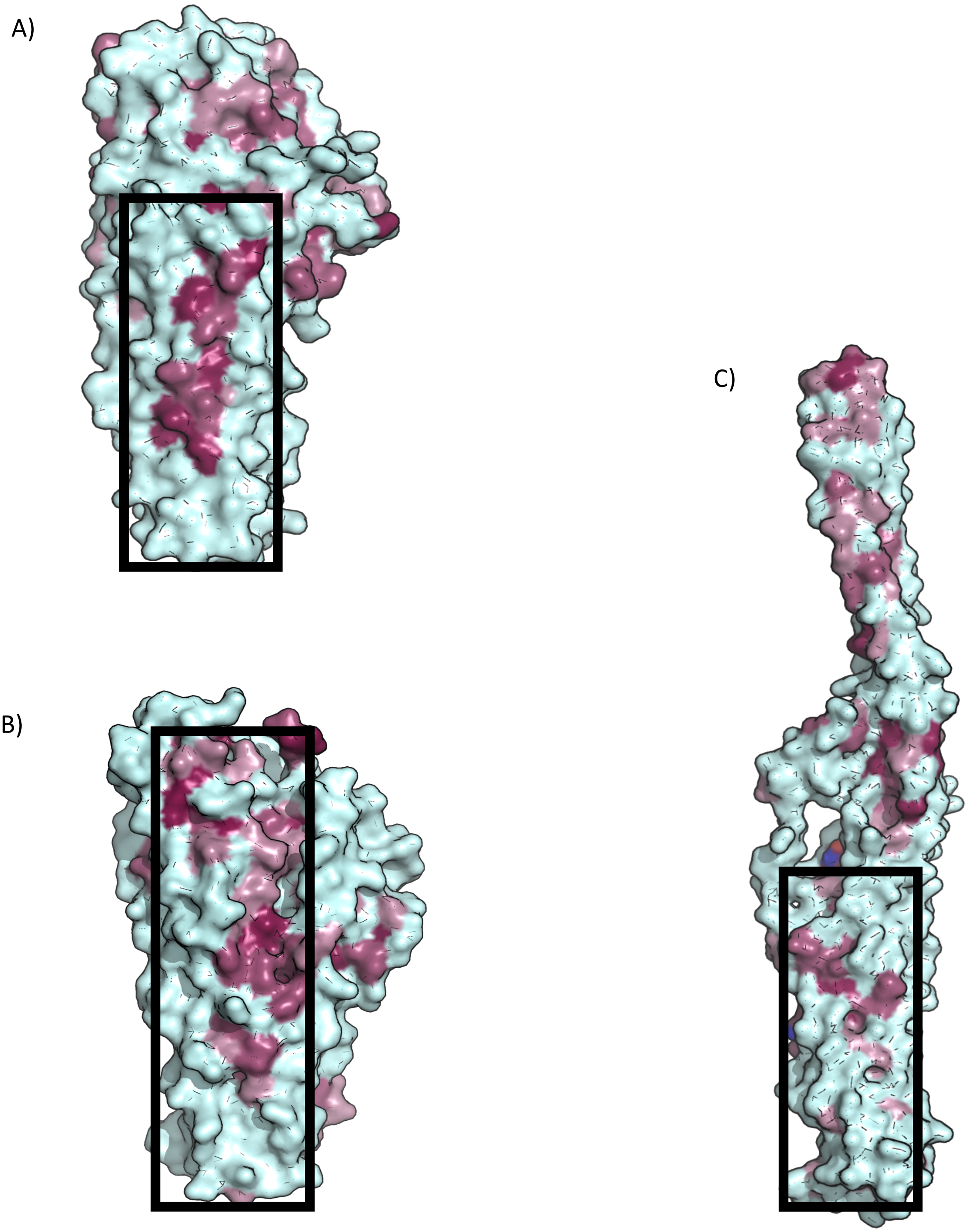

Supplementary Figure 5. Surface representation of (A) SmhA, (B) SmhB, and (C) AhlBT2. Family conserved residues are shown in purple. Black boxes outline predicted binding surfaces between the Smh components for (A) SmhA to SmhB, (B) SmhB to SmhC, (C) SmhB to SmhA.

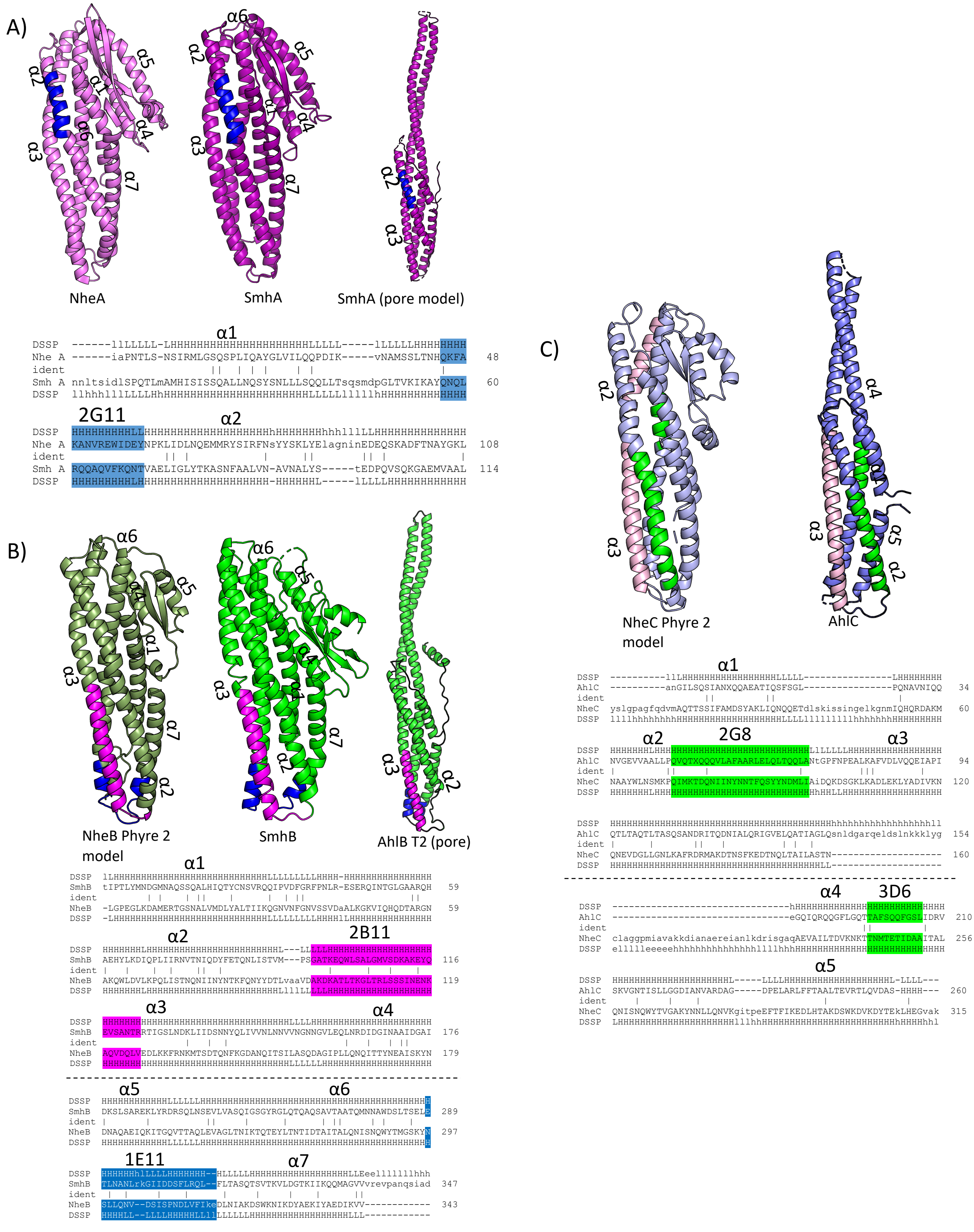

Supplementary Figure 6 Mapping of Nhe antibody binding sites onto Smh and Ahl proteins (A) Structures of NheA (left) showing the 2G11 (blue) Mab binding site (identified by Didier et al [2] and SmhA (right) with NheA 2G11 Mab site mapped onto structure based on Dali [1] structural alignment (below). (B) Phyre 2 [3] model of NheB (left) showing 2B11 (pink) and 1E11 (blue) Mab binding sites and structures of SmhB soluble and pore forms, with the NheB Mab sites mapped onto these structures based on Dali structural alignments (below). (C) Phyre 2 model of NheC (left), showing 2G8 and 3D6 Mab binding sites (green), which do not disrupt complex formation with predicted binding site for the B component identified by Heilkenbrinker [4] (pink) and the structure of AhlC (Right) with NheC Mab sites mapped onto the structure based on Dali structural alignment (below).

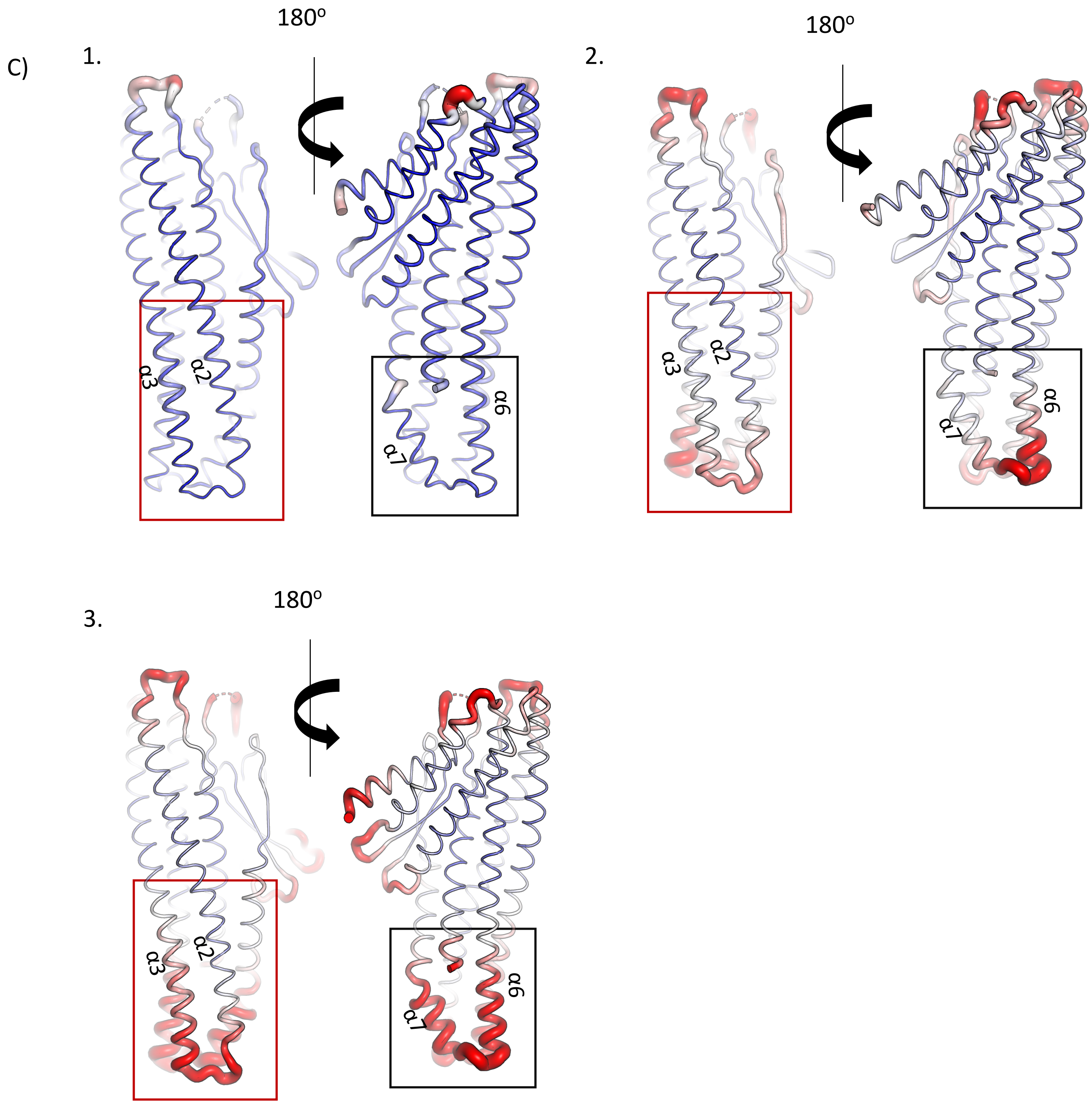

Supplementary Figure 7. Flexible regions of SmhB. Variations in B-factor (blue – low B-factor, to red - High B-factor) between monomers of different crystal structures of the soluble form of SmhB**.** An increasing B-factor can be seen at residue 85-124 (red box) and 280-320 (black box) from panels A-C. (A) Crystal form 1, chain A (PDB code 6ZZ5). Crystal form 2 (PDB code 6ZZH) chain A (B) and chain B (C) .

Supplementary information References

1. Holm, L. & Laakso, L. M. Dali server update. *Nucleic Acids Res.* **44**, 351–355 (2016).
2. Didier, A., Dietrich, R. & Märtlbauer, E. Antibody Binding Studies Reveal Conformational Flexibility of the Bacillus cereus Non-Hemolytic Enterotoxin (Nhe) A-Component. *PLoS One* **11**, e0165135 (2016).
3. Kelley, L. A., Mezulis, S., Yates, C. M., Wass, M. N. & Sternberg, M. J. E. The Phyre2 web portal for protein modeling, prediction and analysis. *Nat. Protoc.* **10**, 845–858 (2015).
4. Heilkenbrinker, U. *et al.* Complex Formation between NheB and NheC Is Necessary to Induce Cytotoxic Activity by the Three-Component Bacillus cereus Nhe Enterotoxin. *PLoS One* **8**, e63104 (2013).
